# Evidence-Based Mental Health at Scale: Benchmarking Retrospective Cohort Study of a Digital Employee Benefits Program for Depression and Anxiety

**DOI:** 10.2196/72999

**Published:** 2025-10-29

**Authors:** Emily J Ward, Matt Hawrilenko, Millard Brown, Adam M Chekroud

**Affiliations:** 1 Spring Health New York, NY United States; 2 Department of Psychiatry Yale School of Medicine Yale University New Haven United States

**Keywords:** depression and anxiety, measurement-based care, employer-sponsored mental health, clinical outcome evaluation, digital mental health benefit

## Abstract

**Background:**

Depression and anxiety affect millions worldwide; yet, many people face barriers to timely and effective mental health care, underscoring the need for scalable, high-quality interventions.

**Objective:**

This study aimed to evaluate the clinical effectiveness and quality of a centralized, employer-sponsored mental health program in treating depression and anxiety during a period of rapid growth in access to mental health care.

**Methods:**

This retrospective cohort study included participants using a digital mental health benefit (Spring Health), sponsored by 589 US employers from 2021 to 2024. Participants had access to therapists, psychiatrists, and care navigators. Primary measures were clinical effectiveness (treatment duration, Patient Health Questionnaire 9-item depression scale, Generalized Anxiety Disorder 7-item Scale) and clinical outcomes (reliable change, recovery, and remission). Outcomes were benchmarked to meta-analytic results of evidence-based therapy.

**Results:**

A total of 52,929 adult participants started therapy from among 6868 providers during the study period, scored positive for depression or anxiety, and had at least one mental health assessment before and during treatment. Depression symptoms decreased with each log-day in treatment, resulting in a total reduction of 6.91 (95% CI –6.84 to –6.98) points at one-week post treatment, corresponding to a large effect size (*d*=1.61; 95% CI 1.60-1.63), significantly greater than the meta-analytic pre-post benchmark for psychotherapy (effect size difference=0.13, *z*=15.6, *P*<.001). Anxiety symptoms also decreased, resulting in a total reduction of 6.01 points (95% CI –5.95 to –6.08), corresponding to a large effect size (*d*=1.82; 95% CI 1.80 to 1.84), significantly greater than the meta-analytic benchmarks (effect size difference=0.64, *z*=61.9, *P*<.001). White participants and participants of color had similar outcomes. Logistic regression showed 92.3% (95% CI 92.0% to 92.5%) of participants’ symptoms (in depression or anxiety) reliably improved, and 61.7% (95% CI 61.1% to 62.4%) achieved remission by 1-week posttreatment.

**Conclusions:**

Among a large and diverse sample, using a digital mental health benefit with a centralized system of care produces clinical outcomes in depression and anxiety significantly greater than what is typically observed through meta-analyses of psychotherapy. By using data to monitor, incentivize, and improve quality of care, the clinical outcomes outperform or equal benchmarks as a growing number of individuals across race, gender, and age access mental health care.

## Introduction

Depression and anxiety affect over 280 million people worldwide [1,2], with over 9% of Americans having depression and 18% having anxiety [3]. Although many barriers to mental health care continue to prevent people from getting the type of treatment and quality of treatment they need [4-6], mental health has become a priority across public and private sectors. As employee-benefit packages providing mental health care have proliferated [7], the focus is increasingly on optimizing care: delivering the right care at the right time to ensure positive clinical outcomes [8,9] and financial return on investment through reduced health care spending [8,10] and increased workplace productivity [8,11].

However, simply offering mental health services does not guarantee quality care, and scaled programs without methods to promote and monitor evidence-based treatment can undermine their potential impact [12]. Measurement-based care [13,14]—regularly assessing symptom change and clinical progress—can help address this challenge by ensuring treatment is continuously evaluated for effectiveness. At scale, large data sets offer unprecedented opportunities to evaluate outcomes across diverse populations [15], track provider outcomes, and refine patient-provider matching so that each patient’s clinical profile and treatment preferences are respected. But any mental health solution at scale must first ensure an adequate number of qualified mental health professionals to meet demand and to have positive clinical outcomes.

One promising strategy for promoting accessible, high-quality care is to build broad provider networks that offer a variety of empirically supported treatments. On one hand, a broad provider network could increase treatment accessibility and patient satisfaction, potentially leading to better outcomes. On the other hand, this flexibility necessitates evaluating treatment outcomes. A recent review of digital mental health solutions demonstrated that programs that offer a variety of treatment options show strong positive outcomes, such as the percentage of patients seeing statistically significant improvements in depression, ranging from mid-60% to low-80% [16]. The outcomes of these programs should also be compared with established clinical benchmarks, such as those produced by specific treatment modalities such as cognitive behavioral therapy (CBT) (eg, Cohen *d* or equivalent metrics of 0.63 for depression [17] and 0.51 for anxiety [18]) to ensure the sustainability of high quality outcomes within diverse service settings and among heterogeneous populations.

This retrospective cohort study aimed to evaluate the therapeutic effectiveness of an employer-sponsored mental health program serving 53,000 patients with depression and anxiety through a network of more than 6800 providers. Leveraging the scale of these patient-provider pairs, we aimed to benchmark the program’s effectiveness overall, explore outcomes among different demographic subgroups, and determine how effect sizes changed as the program scaled. By capitalizing on extensive measurement data, we provide insights into how large-scale programs can preserve high-quality care through timely access, tailored provider matching, and rigorous monitoring of clinical outcomes.

## Methods

### Ethical Considerations

This retrospective cohort study was approved by the Yale Institutional Review Board (protocol ID: 2000029276) and classified as not involving research with human participants. Informed consent was not required, as the data were anonymized prior to analysis. The research adhered to the Strengthening the Reporting of Observational Studies in Epidemiology (STROBE) guidelines for observational studies. While some authors had access to identifiable information during and after data collection, all analyses were performed using deidentified data. The privacy and confidentiality of research participants’ data and identity was maintained. Participants did not receive compensation.

### Program Design

Data came from an employer-sponsored digital mental health benefit (Spring Health; Spring Care Inc). The program incorporates evidence-based components to increase access to and use of mental health care as follows.

After completing an initial assessment (see Assessments), participants received a personalized care plan and were able to schedule appointments. Care recommendations ranged from using a library of digital self-help modules (eg, mindfulness exercises, CBT skill-builders) or coaching for low to low-moderate risk concerns. For moderate to high risk concerns, participants could engage in unlimited, no-cost video appointments with care navigators who assisted with finding appropriate care, or could browse the network of clinical care providers and self-schedule appointments directly. Regardless of risk level, all participants had access to free or low-cost access to psychotherapy (with additional sessions offered as an in-network benefit covered by their health plan) and medication management through video or in-person sessions. Providers listed their clinical specialties and modalities on their profiles, with the predominant modality being CBT. Across these different care options, the platform delivers care through a centralized data system in which all provider notes, patient data, assessment results, patient scheduling, and payment are integrated into one system. In the centralized system of care, average provider availability was one day nationwide. Patients could directly book their chosen provider’s appointment. Measurement‑based care was implemented, and incentive structures rewarded and retained top‑performing providers.

Therapists held master or doctoral-level licenses, and all medication providers were either physicians or psychiatric nurse practitioners. All clinicians had at least 3 years of postsupervision experience before joining the network. A total of 6868 providers provided care to participants in the study.

To lower the financial barriers to accessing care, participants could book an unlimited number of free appointments with a care navigator and had access to a varying number of employer-sponsored sessions with a therapist or medication provider (median 12, IQR 8-14). Additional sessions with the same providers could be continued with copays and deductibles according to behavioral health plan selection and coverage, often as in-network coverage with platform providers for over 50% of cases.

### Setting

Treatment was primarily delivered through videoconferencing, and assessments were collected using the program platform. The study period was from January 1, 2021, to June 1, 2024, and only the first epoch of care (no more than 6 months could elapse between therapy sessions) was considered. Appointment and assessment data were collected up to 6 months after the first therapy appointment. Final assessments that occurred beyond 1 SD of the average interval from the final appointment were excluded prior to analyses (ie, within about 35 [–18 days before end of treatment + 53] days of the final appointment). The final study size was determined by how many participants met the inclusion criteria (see Participants).

### Participants

Participants were employees and dependents eligible for the benefit during the study period. Participants were included if they took an assessment and started treatment within the study period, if they were located in the United States, were older than 18 years, had a baseline assessment within 30 days of starting treatment or before the second appointment if they screened positive for depression or anxiety (Patient Health Questionnaire 9-item Scale [PHQ-9]≥10 or Generalized Anxiety Disorder 7-item Scale [GAD-7]≥10), and if they had an additional assessment after treatment began. This criterion aimed to capture a broader real-world population engaging with the service while still ensuring a pretreatment baseline and at least one follow-up for outcome measurement. Participants must have remained eligible for the benefit for at least 6 months after starting, across which period data were collected. Participants did not receive a monetary reward.

### Assessments

The program included a digital mental health assessment tool, which enabled a proactive symptom monitoring system to support measurement-based care. Within the digital platform, individuals completed a series of questionnaires to identify common mental health difficulties (such as stress, anxiety, sleeping, eating, or relationship issues). They completed the PHQ-9 [19,20] for depression and additional self-report questionnaires based on the mental health issues identified by the participant. For those with anxiety, this included the GAD-7 [21]. The cut-off for a positive depression screening was PHQ-9≥10; for a positive anxiety screening, it was GAD-7≥10. Participants were reminded by email every 2 to 4 weeks while in care to complete follow-up assessments, but all assessments were optional, and participants were not required to complete an assessment at the end of treatment.

### Measures

#### Depression and Anxiety

Depression symptoms were measured with the PHQ-9 (range 0-27), consisting of 9 items assessing the frequency of a range of depression symptoms. Anxiety symptoms were measured with the GAD-7 (range, 0-21), consisting of 7 items assessing the frequency of a range of anxiety symptoms. Both PHQ-9 and GAD-7 items are scored 0-3 (not at all to nearly every day). PHQ-9 and GAD-7 scores were outcome variables for assessing symptom change (continuous outcomes), reliable improvement [22] (5-point decrease in the PHQ-9 [19] and 4-point decrease in the GAD-7), and reliable improvement with recovery (both reliable improvement and ending in the subclinical range [20], corresponding to a PHQ-9 score <10 or a GAD-7 score of <10) and remission (ending with PHQ-9 and GAD-7 scores <5). Depression and anxiety symptoms were modeled separately.

#### Factors Associated With Clinical Improvement

The primary outcome was change in clinical symptoms over time (days in treatment, measured from the start of therapy [*t*=0], estimated at 1-week posttreatment, corresponding to the average duration of care across all patients plus one week [*t*=95.4 average duration + 7 days], and at the end of 6 months [*t*=180 days]). Demographics (age, race, and gender) were collected from employer census files or when individuals enrolled in the program. The program did not compel individuals to disclose personal information, such as their race or gender, and those choosing to share such information did so through free-response, thereby self-endorsing their own identities, which were categorized for data analysis or marked as not available (“Missing” in Table 1). No attempts were made to “predict” missing demographic information using any identifying personal information.

**Table 1:** Descriptive characteristics of the study sample.

Characteristics			Overall (N=52,929)
**Race, n (%)**			
	White	9940 (61.2)	
	Asian	1166 (7.2)	
	Hispanic or Latino	1840 (11.3)	
	Two or more races/ethnicities	456 (2.8)	
	Black or African American	2737 (16.8)	
	American Indian or Alaska Native	111 (0.7)	
	Total	16,250 (30.7% of total)	
	Missing	36,679 (69.3% of total)	
**Gender, n (%)**			
	Man	2436 (24.3)	
	Woman	7341 (73.3)	
	Non-Binary	233 (2.3)	
	Total	10,010 (18.9% of total)	
	Missing	42,919 (81.1% of total)	
**Age group (years), n (%)**			
	18 to 30	13,751 (26.0)	
	30 to 50	30,117 (56.9)	
	50 to 65	8215 (15.5)	
	>65	846 (1.6)	
	Total	52,929 (100% of total)	
**Age (years)**			
	Mean (SD)	37.9 (11.2)	
	Median (Min-Max)	36.0 (18.0-88.0)	
**Baseline PHQ-9^a^ (positive screening)**			
	Mean (SD)	15.4 (4.29)	
	Median (Min-Max)	15.0 (10.0-27.0)	
	N (%)	42,630 (80.5)	
**Baseline GAD-7^b^ (positive screening)**			
	Mean (SD)	14.5 (3.31)	
	Median (Min-Max)	14.0 (10.0-21.0)	
	N (%)	39,860 (75.3)	

^a^PHQ-9: Patient Health Questionnaire 9-item Scale.

^b^GAD-7: Generalized Anxiety Disorder 7-item Scale.

#### Missing Data

**Figure 1:**
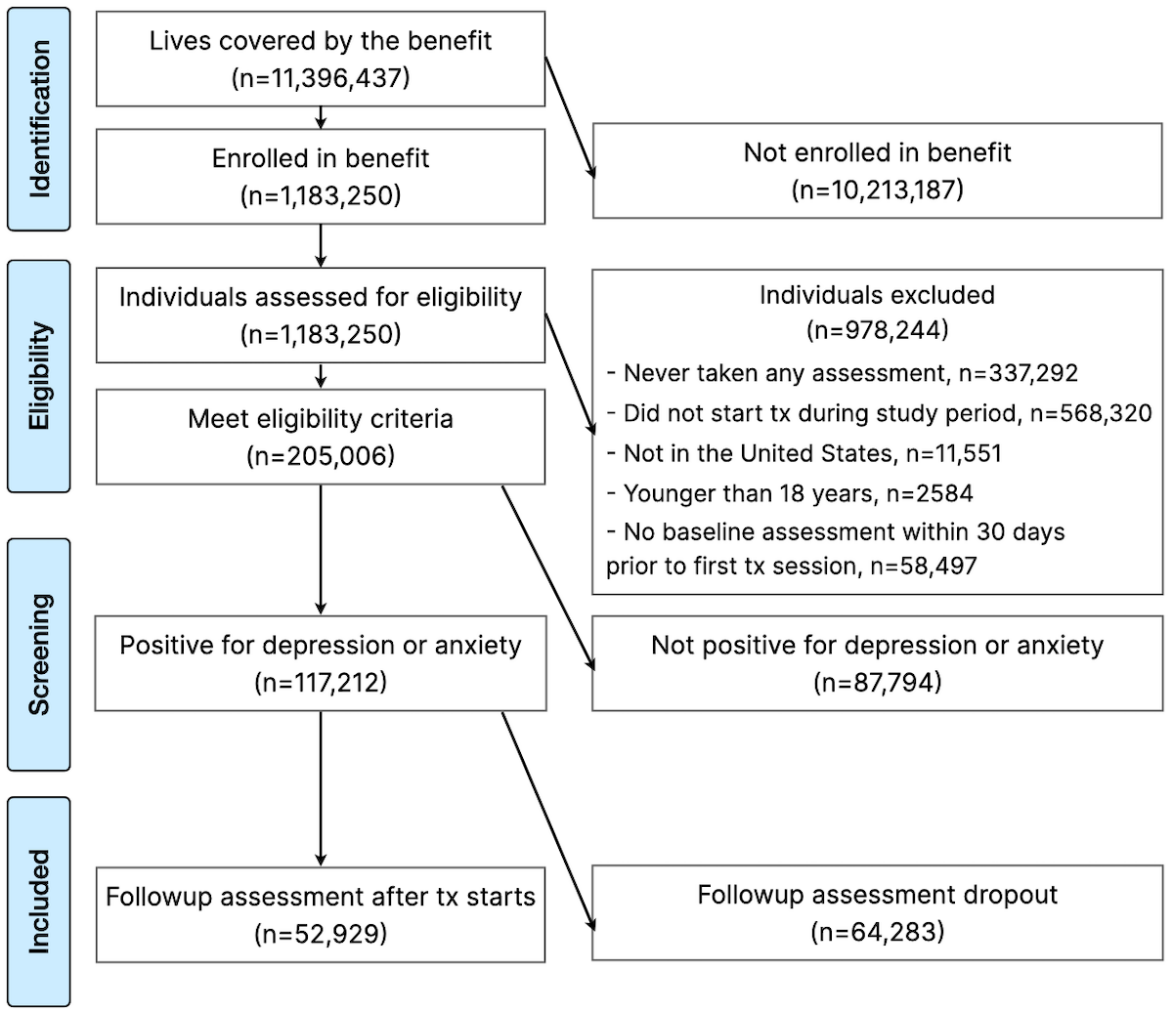
Diagram depicting patient participant flow through the study from identification to study inclusion.

This study had 2 primary types of missing data. The first type of missing data was assessment drop-out, where patients did not complete any follow-up assessment after starting care, excluding 55% (n=64,283) of potential patients (Figure 1). The impact of this missingness was examined through 2 complementary approaches: (1) evaluating how treatment dosage (ie, number of therapy sessions) differed between those with and without follow-up assessments and (2) reweighting the data according to the treatment dosage distribution of those without follow-up assessments. The second type of missing data was demographic data, because neither race nor gender was required by the platform. To ensure our final sample was representative of our treatment-eligible population (those who enrolled in the benefit, n=1,183,250), we compared the racial and gender proportions using a chi-square test. Subgroup analyses are presented in accordance with best practice for equity-focused research [23,24], although due to the extent of missingness of demographic data, these analyses should be considered exploratory.

### Meta-Analytic Benchmarks

Benchmarking single-study findings against meta-analytic estimates is standard practice in psychotherapy and health-services research, and can help improve the quality of interventions [25]. Effect sizes obtained from randomized control trials or meta-analyses have been used to evaluate the effectiveness of natural-setting psychotherapy [26] to large behavioral health agencies [27]. Furthermore, selecting an appropriate benchmark helps rule out improvement driven by extraneous factors or reversion to the mean in naturalistic samples.

In this study, the treatment effect sizes were compared with two types of meta-analytic benchmarks: (1) uncontrolled prepost effect sizes for psychotherapy and (2) the prepost effect size for treatment as usual (TAU). The uncontrolled prepost comparison can show the typical magnitude of within-group improvement that occurs over the course of psychotherapy, while the prepost TAU comparison can show whether an intervention has additional value above what is usually done in routine care (vs waitlist controlled trials often overestimate the effect sizes of psychotherapies [28]). There is heterogeneity across settings, and the meta-analytic effect size was derived from treatments spanning cognitive-behavioral, behavioral activation, interpersonal, third-wave, group, individual, and guided self-help formats. Furthermore, these studies comprised different study designs: most had 2 conditions, a subset had more than 2; about half used a cut-off score, and the rest used a diagnosis; participants came from community recruitment, clinical recruitment, or another method. While each individual study was testing a specific intervention, the meta-analysis summarizes the effect of therapy across all these different designs and interventions. But there are no significant differences between major categories of TAU when compared with psychotherapy conditions in randomized trials [29].

### Data Analyses

We used mixed-effects regression models to estimate clinical outcomes. This approach is the widely accepted, gold-standard methodological approach to analyze outcomes—especially binary outcomes—with attrition and irregularly spaced measurements over time [30-32].

To address repeated observations for the same participants (such as multiple assessments over time), we used a mixed-effects model with repeated observations nested within participants as a random intercept. An identity link was used for continuous outcomes (depression and anxiety) and a logit link for categorical outcomes (reliable change, recovery, and remission). Symptom change, modeled using log-days since the start of treatment, was the main effect of interest. To determine which factors were associated with symptom change, the set of measures described above (age, gender, and race) was included as covariates. To account for the effect of provider on outcomes, provider was included as a random intercept; to investigate different trajectories of improvement at the participant and provider levels, a random slope term (ie, log-days in treatment) was included for both participant and provider.

Total improvement from the start of treatment to the desired endpoint (such as end of treatment or 180 days) was calculated using the delta method [33]. The delta method multiplies the rate of change (ie, the regression coefficient for log-days in treatment) by the desired duration to produce total improvement and standard errors from a function that combines model parameters. It avoids using the last observation carried forward, which has been shown to introduce bias [34,35].

Reliable improvement, recovery, and remission rates were predicted separately using logistic models with log-days since start of treatment as the main effect. Regression coefficients from these models were converted to probabilities to estimate the percentage of participants who showed reliable improvement, recovery, or remission from depression and anxiety symptoms within a modeled margin of error (ie, 95% CIs).

The treatment effect size was quantified using Cohen *d*, calculated by dividing the total treatment improvement in the measure of interest (eg, symptom-point decrease in PHQ-9) by the pre-test SD (eg, SD of PHQ-9 scores at baseline) to avoid a potential impact of care on the posttest SD [36]. There was a correlation (*r*) of 0.36 between PHQ-9 scores at baseline and at treatment duration, and a correlation (*r*) of 0.27 for GAD-7 scores. Effect sizes were categorized using established thresholds of small (*d* <0.50), medium (*d* <0.8), and large (*d* >0.8).

The treatment effect size was compared with meta-analytic benchmarks in two ways: (1) the overall prepost treatment effect was compared with uncontrolled prepost effect sizes for psychotherapy (depression: uncontrolled effect size=1.48 [28] and anxiety: uncontrolled effect size=1.18 [37]) and (2) to adjust for expected improvement in the course of usual care, the prepost effect size for TAU (depression: TAU effect size=0.64 [28] and anxiety: TAU effect size=0.59 [37]) was subtracted from the overall prepost treatment effect. The TAU-adjusted effect size was then compared with benchmark effect sizes for psychotherapy in randomized controlled trials (depression: effect size=0.63 [28] and anxiety: effect size=0.51 [37]) with TAU as the control group.

For example, an overall prepost treatment effect size of 1.50 would be compared with the uncontrolled effect size of 1.48; the TAU-adjusted effect size of 0.87 (1.50-0.64) would be compared with the RCT effect size of 0.63. All effect sizes were compared using a 1-sample z test. Using this TAU-adjustment is a means of approximating the true effect size of the program intervention in the absence of a control group.

To assess changes in effect size as a function of program scale, the difference between treatment effect size and benchmark values was predicted by the number of lives covered (in millions) using a linear regression.

All statistical tests were 2-sided with statistical significance set at α levels of .05. All analyses were conducted in R (version 2024.04.2; Posit, PBC) [38].

## Results

### Participant and Provider Characteristics

A total of 11,396,437 people were covered by the benefit during the study period, and 1,183,250 (10%) people enrolled (Figure 1). These individuals were assessed for eligibility, and 205,006 (17%) people met all eligibility criteria. Of these, 117,212 (57%) people screened positive for depression or anxiety. Finally, 52,929 (45%) people had an additional assessment after treatment began. This follow-up assessment was taken on average 34 (SD 36) days after the start of treatment, 14 (SD 21) days from the most recent session, and 64 (SD 56) days before the final session of treatment. The final assessment was taken on average 68 (SD 54) days after the start of treatment, 19 (SD 24) days from the most recent session, and 27 (SD 41) days before the final session of treatment.

Among the 52,929 participants who met the inclusion criteria (Table 1), their ages ranged from 18 to 88 (mean 37.9, SD 11.2) years. A total of 7341 of 10,010 (73.3%) participants with known gender information were female, and 9940 of 16,250 (61.2%) participants with known race information were White. This final sample of participants reflected 589 different employers. In addition, 42,630 participants screened positive for depression (scores ≥10 points) and 39,860 screened positive for anxiety (scores≥10 points), although not all provided race or gender. Whereas, 29,561 participants screened positive for both depression and anxiety, corresponding to a 55.9% comorbidity rate, similar to the general population [39-41]. Among the participants screening positive, baseline scores were moderate to severe for both PHQ-9 (mean 15.4, SD 4.3) and GAD-7 (mean 14.5, SD 3.3). Participants received care from 6868 providers.

### Overall Clinical Outcomes

During the study period, the average number of business days to the next available appointment was 1.2 (SD 0.4 days. For example, if a patient searched for an appointment on Monday, the next available appointment they could book would be sometime on Tuesday. The average number of therapy sessions was 6.6 (SD 4.7), and the average number of medication management sessions was 2.7 (SD 1.8; 19.7% of participants attended at least one medication management session). Participants spent approximately 3 months in treatment (95.4, SD 58.5 days), with one-week posttreatment defined as 102 days. The average number of total assessments was 3.3 (which included 1 baseline assessment and 2.3 assessments taken after therapy began).

**Figure 2:**
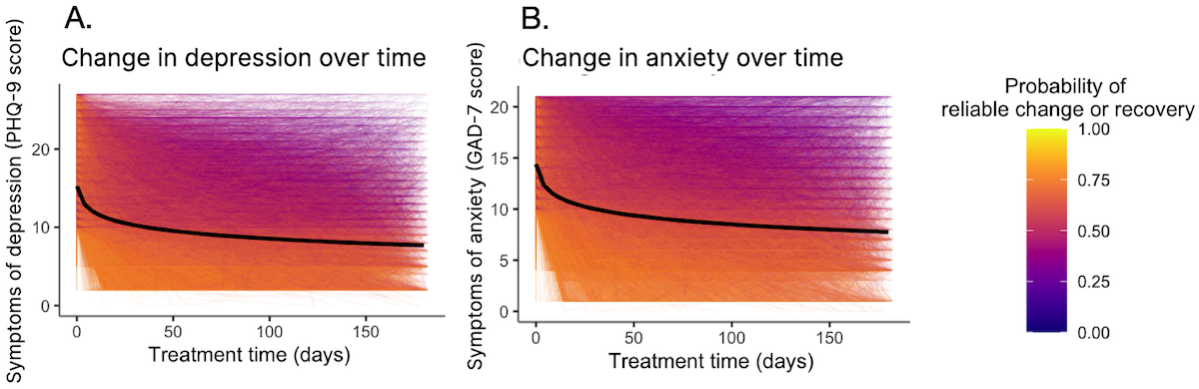
Change in symptoms of (A) depression, as measured using the PHQ-9, and (B) anxiety, as measured using the GAD-7. Overall symptom change is modeled by log-day in treatment and plotted in black on both panels. Each individual trajectory is plotted as an overlay and color-coded according to the probability of achieving either reliable change or recovery. GAD-7: Generalized Anxiety Disorder 7-item Scale; PHQ-9: Patient Health Questionnaire 9-item Scale.

Overall, PHQ-9 scores decreased with each log-day in treatment (*b*=–1.49, SE 0.01; *P*<.001) (Table 2), resulting in a total reduction of 6.91 (95% CI –6.84 to –6.98) points (Figure 2A) at one-week post-average-treatment duration, corresponding to a large effect size (*d*=1.61; 95% CI 1.60-1.63), significantly greater than the uncontrolled prepost effect size for psychotherapy [28] (effect size difference=0.13, *z*=15.6, *P*<.001). When adjusted for the expected effect of TAU, the effect size remained large (*d*=0.97; 95% CI 0.96-0.99) and significantly greater than meta-analytic benchmarks (effect size difference=0.34, *z*=40.6, *P*<.001).

GAD-7 scores also decreased with each log-day in treatment, (*b*=–1.30, SE 0.01; *P*<.001; Table 2), resulting in a total reduction of 6.01 (95% CI –5.95 to –6.08) points (Figure 2B), corresponding to a large effect size (*d*=1.82; 95% CI 1.80-1.84), significantly greater than the uncontrolled prepost effect size for psychotherapy (effect size difference=0.64, *z*=61.9, *P*<.001). When adjusted for the expected effect of TAU, the effect size remained large (*d*=1.23; 95% CI 1.21-1.25) and significantly greater than meta-analytic benchmarks (effect size difference=0.72, *z*=69.7, *P*<.001).

**Figure 3:**
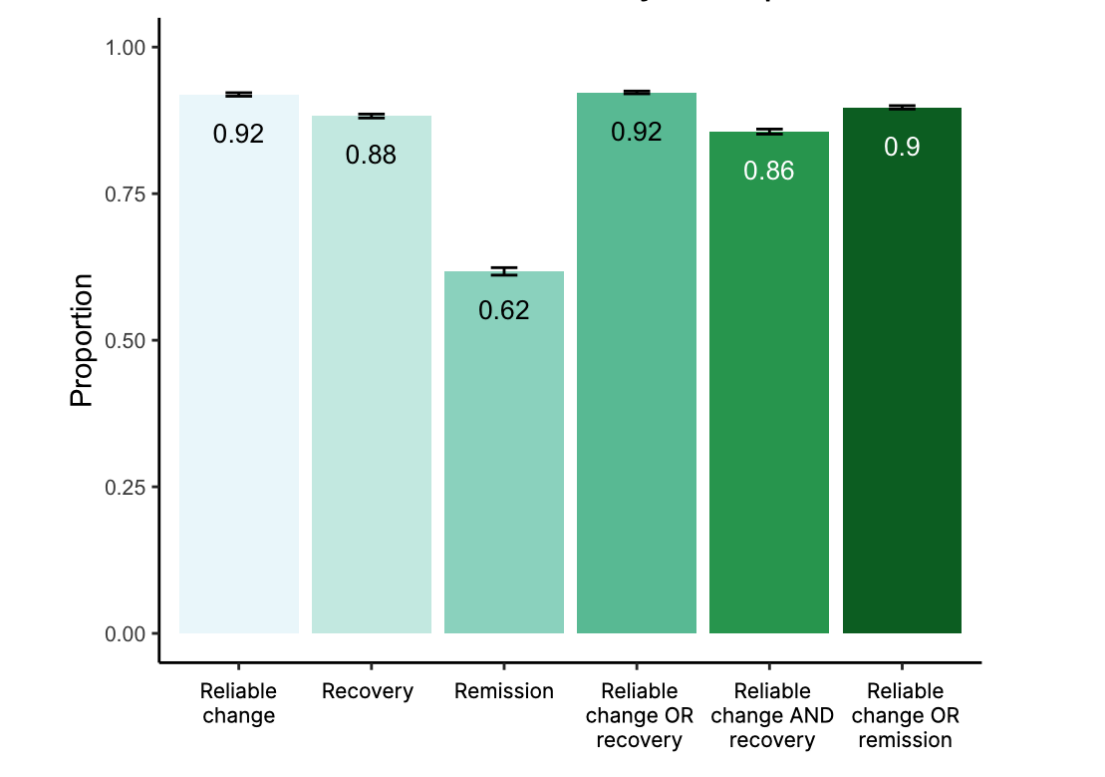
Clinical outcome results from a mixed-effects logistic regression for depression and anxiety. The predicted proportion of participants achieving reliable change (reduction of 5 points on Patient Health Questionnaire 9-item Scale [PHQ-9] or 4 points on Generalized Anxiety Disorder 7-item Scale [GAD-7]), recovery (scores of less than 10 points on PHQ-9 or less than 8 on GAD-7), or remission (scores of less than 5 points on either PHQ-9 or GAD-7) is plotted, along with the predicted proportion achieving combinations of clinical outcomes.

By the time of their final assessment (average of 68 days), 89.0% (n=47,131) of participants reliably improved or recovered as measured directly by change in their assessment scores. By 1-week posttreatment (102 days), results of mixed-effects logistic regression models (Figure 3) indicated that 92.3% of participants reliably improved or recovered (95% CI 92.0%-92.5%) and 61.7% (95% CI 61.1%-62.4%) achieved remission.

**Table 2:** Regression estimates of overall improvement in depression and anxiety symptoms.

					Depression (PHQ-9^a^ score)		Anxiety (GAD-7^b^ score)
**Intercept**							
	Estimates				15.27		14.39
	SE				0.02		0.02
	*P* value				<.001^c^		<.001^c^
**Time in treatment (log-days)**							
	Estimates				–1.49		–1.30
	SE				0.01		0.01
	*P* value				<.001^c^		<.001^c^
**Random effects**							
	σ^2^				12.94		10.79
	**τ_00_**						
		Member_id		7.30		2.28	
		Provider_id		0.29		0.16	
	**τ_11_**						
		Member_id.time in tx		0.57		0.45	
		Provider_id.time in tx		0.03		0.03	
	**ρ_01_**						
			Member_id	0.22		0.94	
			Provider_id	–0.25		–0.28	
	Intraclass correlation				0.56		0.54
	**N**						
			Member_id	42,630		39,860	
			Provider_id	5797		5671	
	Observations				137,607		128,966
	Marginal *R*^2^ / Conditional *R*^2^				0.225/0.658		0.217/0.637

^a^PHQ-9: Patient Health Questionnaire 9-item Scale.

^b^GAD-7: Generalized Anxiety Disorder 7-item Scale.

^c^Significant values (*P*<.05).

### Follow-Up Assessment Sensitivity Analyses

Assessment drop-out—where patients did not complete any follow-up assessment after starting care—was a substantial source of missing data, excluding 55% of potential patients (Figure 1). Several sensitivity analyses were conducted to evaluate how treatment dosage (ie, number of therapy sessions) differed between those with and without follow-up assessments and to reweigh the data according to the treatment dosage distribution of those without follow-up assessments.

Overall, the baseline PHQ-9 and GAD-7 were similar between those who had a follow-up assessment (ie, those in the final sample) and those who did not. However, those who lacked a follow-up assessment attended far fewer total therapy sessions (4.0 sessions vs 6.6 sessions). In fact, 27% of those lacking a follow-up assessment attended only a single session (vs 6% among those with over 2 assessments). To adjust our estimates, we included session count into our outcomes model, using maximum likelihood estimation to obtain an unbiased estimate of overall outcomes even when there are systematically fewer follow-up observations for the group of participants with fewer therapy sessions [42]. We re-estimated the PHQ-9 and GAD-7 models including all participants and added the centered session count as an interaction and found that while people who attended more sessions improved slightly more slowly per unit time (time in tx by number of sessions interaction: *b*=0.01, 95% CI 0.01-0.01, *P*<.001 for both PHQ-9 and GAD-7), the effect is small and the overall pattern of improvement remains robust.

Next, to determine whether the difference in distribution impacted our results, we re-estimated our models weighted according to the session distribution in the excluded group. The rate of depression symptom improvement was faster among the reweighted sample (*b*_reweighted_=–1.55) compared with the unweighted sample (*b*=–1.49, Table 2), with similar results for anxiety improvement (*b*_reweighted_=–1.34 vs *b*=–1.30). These results complement the results that included the number of sessions, suggesting that improvement may be faster when participants attend fewer sessions.

Finally, to ensure that our results were not upwardly biased by long intervals between appointments and assessments, we ran a sensitivity analysis where we restricted the interval to be closely tied in time: we excluded any assessments beyond 1 SD or 2 SD of the mean time between appointments and assessments and re-estimated the clinical improvement models. The rate of depression symptom improvement was faster (*b*_1SD_=–1.54 and *b*_2SD_=–1.56) when the interval was restricted compared with the rate of improvement without any restriction on assessment timing (*b*=–1.49, Table 2). A similar pattern was obtained for anxiety symptom improvement. This suggests that the true clinical outcomes of the program may be faster than what we observe when participants are not required to take assessments tied in time to their appointments.

### Clinical Outcomes by Subgroup

#### Overview

To ensure representativeness, we compared our sample to the treatable population (ie, enrolled in benefits). Our sample differed from the population in race (*χ*^2^_5_=93.6, *P*<.001) and gender (*χ*^2^_2_=173.6, *P*<.001), but the effect sizes were negligible to small (Cohen *w*=0.08; *w*=0.13), reflecting more White and Hispanic patients and more women in the final sample than would be expected from population proportions. However, due to the extent of missingness of demographic data, race and gender subgroup analyses should be considered exploratory.

#### Race

**Figure 4:**
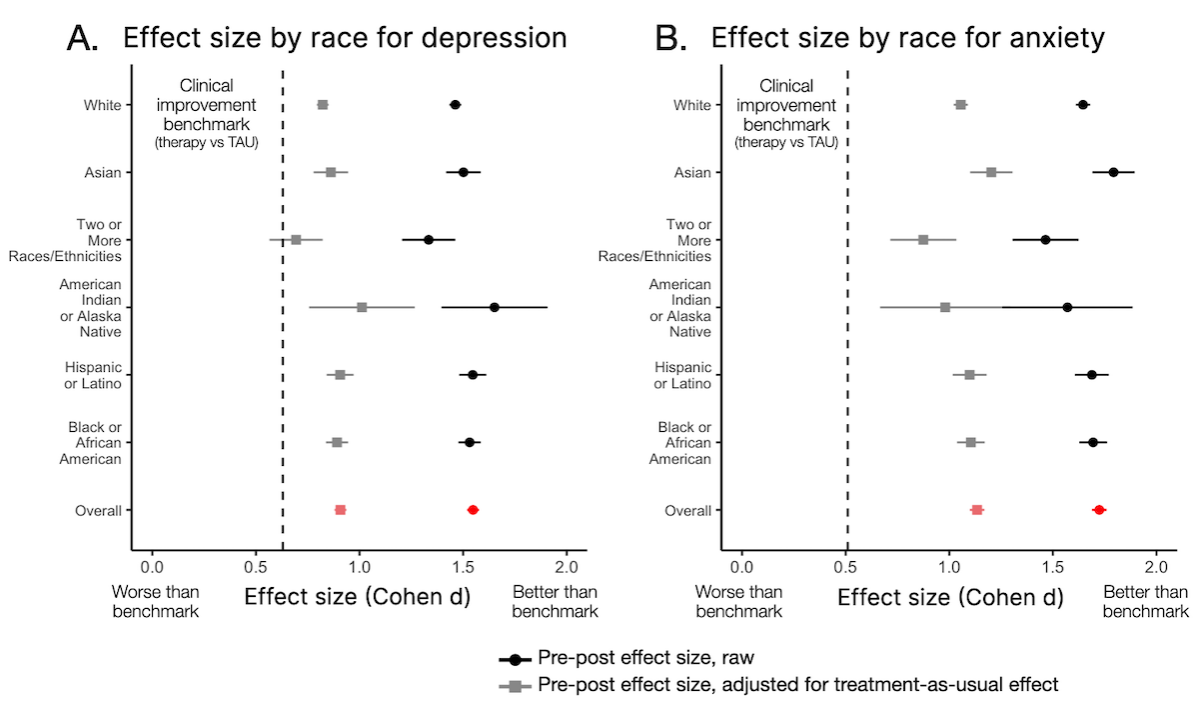
The effect size of clinical improvement for different racial and ethnic minority participants, both for the prepost treatment effect (black) and when corrected for improvements expected due to TAU (gray). The TAU-adjusted effect sizes are plotted relative to meta-analytic effect sizes for psychotherapy versus TAU. (A) Improvement for depression, benchmarked against effect size of 0.63 (Cohen d). (B) Improvement for anxiety, benchmarked against effect size (d) of 0.51. All groups improved at or beyond the benchmarks. Race and ethnicity data were provided by 30.7% of participants. TAU: treatment as usual.

For depression, there was a main effect of race, such that racial minority participants (except Asian) started therapy with higher baseline acuity than White participants (Table 3). Black and Hispanic participants improved faster than White participants (*P*<.011), and Asian participants of 2 or more races improved slower (*P*=.038). Yet, at 1-week post treatment, the overall effect size was large (raw prepost: *d*=1.55; TAU-adjusted *d*=0.91) and all participants’ improvement (*d* values>0.69) was comparable or better than meta-analytic benchmarks (*P*<.3; Figure 4A). For anxiety, only Black participants started therapy with higher baseline acuity than White participants (Table 3). Asian participants improved faster than White participants (*P*=.008), and participants of 2 or more races improved more slowly (*P*=.034). Critically, at 1-week posttreatment, the overall effect size was large (raw prepost: *d*=1.72; TAU-adjusted *d*=1.13) and all participants’ improvement (*d* values>0.87) was comparable or better to meta-analytic benchmarks (*P* values<.01; Figure 4B).

**Table 3:** Regression estimates of improvement in depression and anxiety symptoms by race (race and ethnicity data were provided by 30.7% of participants; Table 1).

				Depression (PHQ-9^a^ score)		Anxiety (GAD-7^b^ score)
**Intercept**						
	Estimates		15.12		14.27	
	95% CI		15.01 to 15.23		14.17 to 14.37	
	*P* value		<.001		<.001	
**Race**						
	**American Indian or Alaska Native**					
		Estimates	1.06		0.57	
		95% CI	0.01 to 2.11		–0.41 to 1.55	
		*P* value	.048		.256	
	**Asian**					
		Estimates	–0.10		0.00	
		95% CI	–0.45 to 0.25		–0.32 to 0.32	
		*P* value	.576		.980	
	**Black or African American**					
		Estimates	0.44		0.23	
		95% CI	0.20 to 0.68		0.01 to 0.45	
		*P* value	<.001		.044	
	**Hispanic or Latino**					
		Estimates	0.37		0.19	
		95% CI	0.09 to 0.65		–0.07 to 0.45	
		*P* value	.009		.154	
	**2 or more races/ethnicities**					
		Estimates	0.65		0.43	
		95% CI	0.12 to 1.17		–0.06 to 0.92	
		*P* value	.015		.083	
**Time in treatment (log-days)**						
	Estimates		–1.37		–1.18	
	95% CI		–1.39 to –1.34		–1.21 to –1.16	
	*P* value		<.001		<.001	
**Race (American Indian or Alaska Native) × Time in treatment**						
	Estimates		–0.17		0.07	
	95% CI		–0.41 to 0.07		–0.16 to 0.29	
	*P* value		.165		.550	
**Race (Asian) × Time in treatment**						
	Estimates		–0.04		–0.10	
	95% CI		–0.12 to 0.04		–0.18 to –0.03	
	*P* value		.376		.008	
**Race (Black or African American) × Time in treatment**						
	Estimates		–0.08		–0.04	
	95% CI		–0.14 to –0.02		–0.10 to –0.01	
	*P* value		.007		.110	
**Race (Hispanic or Latino) × Time in treatment**						
	Estimates		–0.09		–0.04	
	95% CI		–0.15 to –0.02		–0.10 to 0.03	
	*P* value		.011		.258	
**Race (2 or more races/ethnicities) × Time in treatment**						
	Estimates		0.13		0.13	
	95% CI		0.01 to 0.25		0.01 to 0.24	
	*P* value		.038		.034	
**Random effects**						
	σ^2^		15.55		12.99	
	**τ_00_**					
		Member_id	12.80		9.48	
	Intraclass correlation		0.45		0.42	
	**N**					
		Member_id	13,296		12,244	
	Observations		44,628		41,454	
	Marginal *R*^2^ / Conditional *R*^2^		0.206 / 0.565		0.196 / 0.535	

^a^PHQ-9: Patient Health Questionnaire 9-item Scale.

^b^GAD-7: Generalized Anxiety Disorder 7-item Scale.

#### Gender

Men, women, and nonbinary participants’ anxiety scores were not significantly different at the start of treatment, although nonbinary participants started more depressed (Table 4). For both depression and anxiety, nonbinary individuals improved more slowly than men (reference group; Table 4; *P* values<.003), and for anxiety, women also improved more slowly than men (*P*=.003). Critically, at 1 week post treatment, men and women’s TAU-adjusted depression improvement (*d* values>0.77) was comparable or better than meta-analytic benchmarks (*P* values<.001) but nonbinary individuals’ was slightly less (*d* difference=–0.10), although not significantly different from the benchmark (*z*=–1.24, *P*=.22). For anxiety, men, women and nonbinary participants’ TAU-adjusted improvement (*d* values>0.65) was comparable or better than meta-analytic benchmarks (*P*<.20).

**Table 4:** Improvement in depression and anxiety symptoms by gender (gender data were provided by 18.9% of participants; Table 1).

			Depression (PHQ-9^a^ score)	Anxiety (GAD-7^b^ score)
**Intercept**				
	Estimates		15.28	14.50
	95% CI		15.05 to 15.50	14.29 to 14.71
	*P* value		<.001	<.001
**Gender**				
	**Nonbinary**			
		Estimates	0.93	–0.08
		95% CI	0.21 to 1.66	–0.77 to 0.60
		*P* value	.012	.815
	**Woman**			
		Estimates	0.06	–0.17
		95% CI	–0.20 to 0.32	–0.42 to 0.07
		*P* value	.639	.158
**Time in treatment (log-days)**				
	Estimates		–1.31	–1.24
	95% CI		–1.36 to –1.25	–1.29 to –1.19
	*P* value		<.001	<.001
**Gender (Nonbinary) × time in treatment**				
	Estimates		0.24	0.37
	95% CI		0.08 to 0.40	0.21 to 0.53
	*P* value		.003	<.001
**Gender (woman) × time in treatment**				
	Estimates		–0.06	0.09
	95% CI		–0.12 to 0.00	0.03 to 0.14
	*P* value		.065	.003
**Random effects**				
	σ^2^		15.55	12.88
	**τ_00_**			
		Member_id	13.41	9.97
	Intraclass correlation		0.46	0.44
	**N**			
		Member_id	8229	7610
	Observations		29,390	27,395
	Marginal *R*^2^ / Conditional *R*^2^		0.190 / 0.565	0.183 / 0.540

^a^PHQ-9: Patient Health Questionnaire 9-item Scale.

^b^GAD-7: Generalized Anxiety Disorder 7-item Scale.

#### Age

There was no difference in baseline severity for depression between participants aged 30 to 50 years, 50 to 65 years, and 65 years and older (*P* values>.31), but those aged 18 to 30 years were significantly more severe (*P*=.012; Table 5). For both depression and anxiety, younger age groups (30 to 50 years and 18 to 30 years) improved more slowly than the oldest (reference) age group, older than 65 years (Table 5; *P* values<.043). But at 1-week posttreatment, all participants had (TAU-adjusted) improvement greater than meta-analytic benchmarks (*d* values>0.84, *P* values<.001). For anxiety, there was no difference in baseline severity among age groups, and all outperformed meta-analytic benchmarks (*d* values [TAU-adjusted] > 1.1, *P* values<.001).

**Table 5:** Improvement in depression and anxiety symptoms by age group.

			Depression (PHQ-9^a^ score)	Anxiety (GAD-7^b^ score)
**Intercept**				
	Estimates		14.96	14.07
	95% CI		14.58 to 15.33	13.69 to 14.45
	*P* value		<.001	<.001
**Age (years)**				
	**50 to 65**			
		Estimates	0.22	0.20
		95% CI	–0.18 to 0.61	–0.20 to 0.60
		*P* value	.283	.326
	**30 to 50**			
		Estimates	0.22	0.28
		95% CI	–0.16 to 0.60	–0.10 to 0.57
		*P* value	.266	.146
	**18 to 30**			
		Estimates	0.50	0.35
		95% CI	0.11 to 0.88	–0.04 to 0.74
		*P* value	.012	.079
**Time in treatment (log-days)**				
	Estimates		–1.56	–1.37
	95% CI		–1.65 to –1.48	–1.46 to –1.29
	*P* value		<.001	<.001
**Age (50 to 65 years) × time in treatment**				
	Estimates		0.03	0.05
	95% CI		–0.06 to 0.12	–0.04 to 0.14
	*P* value		.528	.311
**Age (30 to 50 years) × time in treatment**				
	Estimates		0.10	0.09
	95% CI		0.02 to 0.19	0.00 to 0.18
	*P* value		.020	.043
**Age (18 to 30 years) × time in treatment**				
	Estimates		0.19	0.17
	95% CI		0.10 to 0.27	0.08 to 0.26
	*P* value		<.001	<.001
**Random effects**				
	σ^2^		15.56	12.84
	**τ_00_**			
		Member_id	12.46	9.15
	Intraclass correlation		0.44	0.42
	**N**			
		Member_id	42,630	39,860
	Observations		137,607	128,966
	Marginal *R*^2^ / Conditional *R*^2^		0.227 / 0.571	0.221 / 0.545

^a^PHQ-9: Patient Health Questionnaire 9-item Scale.

^b^GAD-7: Generalized Anxiety Disorder 7-item Scale.

### Scaling’s Impact on Outcomes

**Figure 5:**
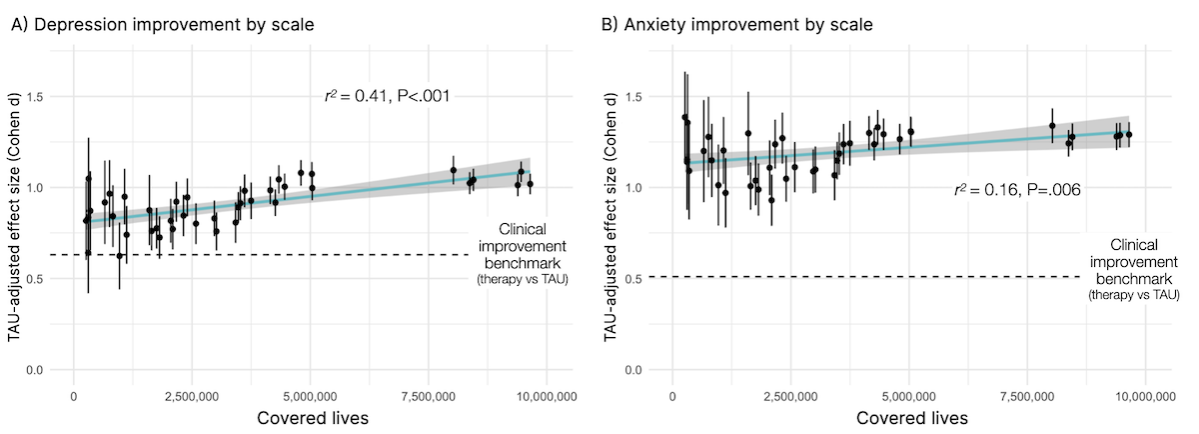
The effect size of clinical improvement (adjusted for improvement expected from TAU) for (A) depression and (B) anxiety as a function of program scale, with x-axis corresponding to how many lives were covered by the program. Improvements were benchmarked against a meta-analytic effect size (Cohen d) of 0.63 for depression and 0.51 for anxiety. The regression estimate is plotted in cyan, showing a significant increase in effect size as a function of scale. TAU: treatment as usual.

A total of 260,548 people were eligible for the benefit at the beginning of the study period, and 9,649,328 were eligible at the end, corresponding to a 37-fold increase in the number of people covered in 3 and a half years. Across the entire study period, 48% of those who enrolled and took an initial assessment started therapy. As the program scaled, this rate of conversion from assessment into care remained stable (*b*=0.00, *P*=.15), indicating treatment accessibility did not decline as a function of scale. Crucially, the effect size for symptom improvement for depression and anxiety increased relative to meta-analytic benchmarks as the program covered and provided treatment to more people ([per million]: depression: *b*=0.03, *t*=5.37, *P*<.001; anxiety: *b*=0.02, *t*=2.92, *P*=.006; Figure 5).

## Discussion

### Principal Findings

This large-scale cohort study—the largest of its kind to date—demonstrates how a comprehensive, employer-sponsored mental health program can facilitate timely access to care and leverage continuous data collection to deliver strong clinical outcomes for both depression and anxiety. Among the nearly 53,000 patients, logistic regression showed 92% of participants experienced reliable symptom improvement or recovery in depression or anxiety, and over 60% achieved remission, with PHQ-9 or GAD-7 scores dropping into the subclinical range, with similar results obtained when calculated directly by change in assessment scores. These results were robust to changes in assessment timing and number of sessions attended, supported through sensitivity analyses. This suggests low upward bias due to exclusion factors, although future work is needed to ensure more participants take reassessments at a regular cadence, which might be accomplished through more visible reminders or by providers during therapy sessions.

Benchmarking analysis found that outcomes met or significantly outperformed established meta-analytic standards for evidence-based treatment, both when considering the uncontrolled prepost effect sizes and when adjusting for expected improvement during TAU. These findings underscore the added value of an integrated, measurement-based approach to care.

Although a substantial portion of participants did not provide their race or gender, the availability of large-scale data enabled exploratory examination of clinical outcomes across diverse subgroups. Racial and ethnic minorities, who often confront systemic barriers to care, leading to lower quality or less evidence-based care [5,6] or provider biases [43], showed comparable rates of improvement to other groups, suggesting that the program provides appropriate support for patients from diverse backgrounds. Likewise, outcomes were similar among different age groups and similar between men and women, although nonbinary participants showed a somewhat smaller magnitude of improvement—an observation that may reflect stressors outside the individual’s control, such as discrimination, stigma, and harassment, as proposed by minority stress theory [44]. Another possible factor could be the extent to which the treatment was tailored to meet the specific needs of nonbinary individuals. Since the current study lacks fine-grain gender data, future research is needed to understand these differences fully. Overall, across all subpopulations, the program outperformed or equaled gold-standard benchmarks [18,45]. Although these results are exploratory and cannot confirm equity, they suggest the program has potential to deliver high-quality care among heterogeneous populations, as well as identifying gaps that future studies or program modifications must address (in particular, how to collect more comprehensive gender and race information without compelling patients to provide it).

Furthermore, these outcomes increased in magnitude as the program expanded, growing its coverage by 37 fold from the beginning of the study period to the end. Program features—including employer-sponsorship, a centralized care platform, care navigation, the ability for patients to rapidly find and choose their therapists, and consistent implementation of measurement-based care—likely contributed to sustaining access and improving clinical effectiveness. For example, therapy sessions were offered for free to patients as part of their benefit package, and the digital platform provided fast access, with an average wait time from assessment to appointment of about one day. Patients could select freely among available providers in the network, and whether they saw a full-time or contracted provider had no impact on their clinical improvement. The enhanced clinical outcomes may be attributed to the combined and interactive effects of multiple care components, rather than to any single intervention, which is typically assessed in benchmarking studies. This suggests that using a flexible range of evidence-based strategies could be crucial for the success of workplace mental health programs.

The current retrospective cohort study evaluated the therapeutic effectiveness of an employer-sponsored mental health program serving 53,000 patients treated by over 6800 providers. Overall, with broad demographic and clinical representation, the results demonstrate that a centralized, measurement-informed system of mental health care delivery can surpass standard benchmarks in improving depression and anxiety symptoms. Moving forward, further research should explore other components, such as how incentives to reward and retain the top-performing providers drive further clinical value.

### Strengths and Limitations

This study has several strengths, such as a diverse and real-world sample, with 39% of participants (of the 16,250 participants who provided their race) being people of color, and a baseline comorbidity rate similar to the general population [39-41], although the results of this study, like other randomized controlled trials of psychotherapy, reflect different demographics than the United States in general (eg, skewing more White and more female). While we investigated broad race, age, and gender identity in our analysis, this approach does not capture the full spectrum of diversity characteristics that can influence therapy outcomes, and a substantial portion of participants opted not to share their race or gender information (69% missing race information; 81% missing gender information). Since missing data may introduce unknown and unmeasured bias into the results and constrain generalizability, the true magnitude of differences between different subgroups may vary, especially if the number of collected assessments differed substantially by demographic group.

Factors such as socioeconomic status, sexual orientation, cultural background, language proficiency, and disability status are integral to understanding individual experiences and treatment effectiveness [43]. As an observational cohort design that only examined outcomes before and after engaging in the program, this study cannot determine a causal link between improvements and engagement in the program. To better understand how measurement-based care contributes to program effectiveness, future interventions should drive higher follow-up assessment rates. Last, this study lacked a control group, although TAU effect sizes were subtracted from all uncontrolled prepost effect sizes to account for this limitation.

### Conclusions

This retrospective cohort study demonstrates that a digital mental health benefit with a centralized system of care produces clinical outcomes in depression and anxiety equivalent to or significantly greater than what is typically observed through meta-analyses of psychotherapy. By using data to monitor, incentivize, and improve the quality of care. Furthermore, the clinical outcomes outperformed or equaled gold-standard benchmarks during a period of rapid growth in access to mental health care.

## Abbreviations

CBT: cognitive behavioral therapy
GAD-7: Generalized Anxiety Disorder 7-item Scale
PHQ-9: Patient Health Questionnaire 9-item Scale
STROBE: Strengthening the Reporting of Observational Studies in Epidemiology
TAU: treatment as usual

## Data Availability

The datasets generated or analyzed during this study are not publicly available because this study comprises personal health information that is considered highly sensitive and confidential. These data are available upon request for researchers based on compliance with legal, regulatory, confidentiality, and patient privacy requirements (please contact the Yale Human Research Protection Program with requests [HRPP@Yale.edu]).

## References

[1] (2024). Depressive disorder (depression). World Health Organization.

[2] Ferrari AJ, Charlson FJ, Norman RE, Flaxman AD, Patten SB, Vos T, Whiteford HA (2013). The epidemiological modelling of major depressive disorder: application for the global burden of disease study 2010. PLoS One.

[3] (2024). Mental health disorder statistics. Johns Hopkins Medicine.

[4] Andrade LH, Alonso J, Mneimneh Z, Wells JE, Al-Hamzawi A, Borges G, Bromet E, Bruffaerts R, de Girolamo G, de Graaf R, Florescu S, Gureje O, Hinkov HR, Hu C, Huang Y, Hwang I, Jin R, Karam EG, Kovess-Masfety V, Levinson D, Matschinger H, O'Neill S, Posada-Villa J, Sagar R, Sampson NA, Sasu C, Stein DJ, Takeshima T, Viana MC, Xavier M, Kessler RC (2014). Barriers to mental health treatment: results from the WHO world mental health surveys. Psychol Med.

[5] Primm AB, Vasquez MJT, Mays RA, Sammons-Posey D, McKnight-Eily LR, Presley-Cantrell LR, McGuire LC, Chapman DP, Perry GS (2010). The role of public health in addressing racial and ethnic disparities in mental health and mental illness. Prev Chronic Dis.

[6] Wang PS, Berglund P, Kessler RC (2000). Recent care of common mental disorders in the United States : prevalence and conformance with evidence-based recommendations. J Gen Intern Med.

[7] (2024). The 2024 NAMI workplace mental health poll. NAMI.

[8] Bondar J, Babich Morrow C, Gueorguieva R, Brown M, Hawrilenko M, Krystal JH, Corlett PR, Chekroud AM (2022). Clinical and financial outcomes associated with a workplace mental health program before and during the COVID-19 pandemic. JAMA Netw Open.

[9] Ward EJ, Fragala MS, Birse CE, Hawrilenko M, Smolka C, Ambwani G, Brown M, Krystal JH, Corlett PR, Chekroud A (2023). Assessing the impact of a comprehensive mental health program on frontline health service workers. PLoS One.

[10] Hawrilenko M, Smolka C, Ward E, Ambwani G, Brown M, Mohandas A, Paulus M, Krystal J, Chekroud A (2025). Return on investment of enhanced behavioral health services. JAMA Netw Open.

[11] de Oliveira C, Saka M, Bone L, Jacobs R (2023). The role of mental health on workplace productivity: a critical review of the literature. Appl Health Econ Health Policy.

[12] Proctor EK, Landsverk J, Aarons G, Chambers D, Glisson C, Mittman B (2009). Implementation research in mental health services: an emerging science with conceptual, methodological, and training challenges. Adm Policy Ment Health.

[13] Lewis CC, Boyd M, Puspitasari A, Navarro E, Howard J, Kassab H, Hoffman M, Scott K, Lyon A, Douglas S, Simon G, Kroenke K (2019). Implementing measurement-based care in behavioral health: a review. JAMA Psychiatry.

[14] Scott K, Lewis CC (2015). Using measurement-based care to enhance any treatment. Cogn Behav Pract.

[15] Khan BN, Liu RH, Chu C, Bolea-Alamañac B, Nguyen M, Thapar S, Fanaieyan R, Leon-Carlyle M, Tadrous M, Kurdyak P, O'Riordan A, Keresteci M, Bhattacharyya O (2024). Reach, uptake, and psychological outcomes of two publicly funded internet-based cognitive behavioural therapy programs in Ontario, Canada: an observational study. Int J Ment Health Syst.

[16] (2025). Virtual solutions for depression and anxiety. Peterson Health Technology Institute.

[17] Cuijpers P, Harrer M, Miguel C, Ciharova M, Karyotaki E (2025). Five decades of research on psychological treatments of depression: a historical and meta-analytic overview. Am Psychol.

[18] Papola D, Miguel C, Mazzaglia M, Franco P, Tedeschi F, Romero SA, Patel AR, Ostuzzi G, Gastaldon C, Karyotaki E, Harrer M, Purgato M, Sijbrandij M, Patel V, Furukawa TA, Cuijpers P, Barbui C (2024). Psychotherapies for generalized anxiety disorder in adults: a systematic review and network meta-analysis of randomized clinical trials. JAMA Psychiatry.

[19] Löwe B, Unützer J, Callahan CM, Perkins AJ, Kroenke K (2004). Monitoring depression treatment outcomes with the patient health questionnaire-9. Med Care.

[20] Kroenke K, Spitzer RL, Williams JBW, Löwe B (2010). The patient health questionnaire somatic, anxiety, and depressive symptom scales: a systematic review. Gen Hosp Psychiatry.

[21] Spitzer RL, Kroenke K, Williams JBW, Löwe B (2006). A brief measure for assessing generalized anxiety disorder: the GAD-7. Arch Intern Med.

[22] Jacobson NS, Truax P (1991). Clinical significance: a statistical approach to defining meaningful change in psychotherapy research. J Consult Clin Psychol.

[23] Lee KJ, Tilling KM, Cornish RP, Little RJA, Bell ML, Goetghebeur E, Hogan JW, Carpenter JR, STRATOS initiative (2021). Framework for the treatment and reporting of missing data in observational studies: the treatment and reporting of missing data in observational studies framework. J Clin Epidemiol.

[24] Flanagin A, Frey T, Christiansen SL, AMA Manual of Style Committee (2021). Updated guidance on the reporting of race and ethnicity in medical and science journals. JAMA.

[25] Lueger R, Barkham M (2010). Using benchmarks and benchmarking to improve quality of practice and services. Developing and Delivering Practice-Based Evidence: A Guide for the Psychological Therapies.

[26] Minami T, Wampold BE, Serlin RC, Hamilton EG, Brown GS(, Kircher JC (2008). Benchmarking the effectiveness of psychotherapy treatment for adult depression in a managed care environment: a preliminary study. J Consult Clin Psychol.

[27] Reese RJ, Duncan BL, Bohanske RT, Owen JJ, Minami T (2014). Benchmarking outcomes in a public behavioral health setting: feedback as a quality improvement strategy. J Consult Clin Psychol.

[28] Cuijpers P, Miguel C, Harrer M, Ciharova M, Karyotaki E (2024). The overestimation of the effect sizes of psychotherapies for depression in waitlist controlled trials: a meta-analytic comparison with usual care controlled trials. Epidemiol Psychiatr Sci.

[29] Cuijpers P, Quero S, Papola D, Cristea IA, Karyotaki E (2021). Care-as-usual control groups across different settings in randomized trials on psychotherapy for adult depression: a meta-analysis. Psychol Med.

[30] Gibbons RD, Hedeker D, Elkin I, Waternaux C, Kraemer H C, Greenhouse J B, Shea M T, Imber S D, Sotsky S M, Watkins J T (1993). Some conceptual and statistical issues in analysis of longitudinal psychiatric data. application to the NIMH treatment of depression collaborative research program dataset. Arch Gen Psychiatry.

[31] Diggle P (2002). Analysis of Longitudinal Data.

[32] Fitzmaurice GM, Laird NM, Ware JH Applied longitudinal analysis. Wiley Online Library.

[33] Oehlert GW (1992). A note on the Delta method. Am Stat.

[34] Kenward MG, Molenberghs G (2009). Last observation carried forward: a crystal ball?. J Biopharm Stat.

[35] Jansen I, Beunckens C, Molenberghs G, Verbeke G, Mallinckrodt C (2006). Analyzing incomplete discrete longitudinal clinical trial data. Statist. Sci.

[36] Harrer M, Cuijpers P, Furukawa T, Ebert D (2021). Doing Meta-Analysis with R: A Hands-On Guide.

[37] Hofmann SG, Kasch C, Reis A (2025). Effect sizes of randomized-controlled studies of cognitive behavioral therapy for anxiety disorders over the past 30 years. Clin Psychol Rev.

[38] R Core Team (2022). R: a language and environment for statistical computing. R Foundation for Statistical Computing.

[39] Brown TA, Campbell LA, Lehman CL, Grisham JR, Mancill RB (2001). Current and lifetime comorbidity of the DSM-IV anxiety and mood disorders in a large clinical sample. J Abnorm Psychol.

[40] Kessler RC, Berglund P, Demler O, Jin R, Koretz D, Merikangas KR, Rush AJ, Walters EE, Wang PS, National Comorbidity Survey Replication (2003). The epidemiology of major depressive disorder: results from the national comorbidity survey replication (NCS-R). JAMA.

[41] Steffen A, Nübel J, Jacobi F, Bätzing J, Holstiege J (2020). Mental and somatic comorbidity of depression: a comprehensive cross-sectional analysis of 202 diagnosis groups using German nationwide ambulatory claims data. BMC Psychiatry.

[42] Hedeker D, Gibbons RD (1997). Application of random-effects pattern-mixture models for missing data in longitudinal studies. Psychol Methods.

[43] Huey S, Park A, Galán CA, Wang C (2023). Culturally responsive cognitive behavioral therapy for ethnically diverse populations. Annu Rev Clin Psychol.

[44] Frost DM, Meyer IH (2023). Minority stress theory: application, critique, and continued relevance. Curr Opin Psychol.

[45] Cuijpers P, van Straten A, Andersson G, van Oppen P (2008). Psychotherapy for depression in adults: a meta-analysis of comparative outcome studies. J Consult Clin Psychol.

